# 3DStructGen: an interactive web-based 3D structure generation for non-periodic molecule and crystal

**DOI:** 10.1186/s13321-020-0411-2

**Published:** 2020-01-23

**Authors:** Pin Chen, Yu Wang, Hui Yan, Sen Gao, Zexin Xu, Yangzhong Li, Qing Mo, Junkang Huang, Jun Tao, GeChuanqi Pan, Jiahui Li, Yunfei Du

**Affiliations:** 1 National Supercomputer Center in Guangzhou, School of Data and Computer Science, Sun Yat-sen University, 132 East Circle at University City, Guangzhou, 510006 China; 2 Department of Information Systems, Business Statistics and Operations Management, Business School, Hong Kong University of Science and Technology, Clear Water Bay, Kowloon, Hong Kong, China

**Keywords:** Visualization, HTML5, Structure editor, 3D

## Abstract

**Background:**

The increasing number of organic and inorganic structures promotes the development of the “Big Data” in chemistry and material science, and raises the need for cross-platform and web-based methods to search, view and edit structures. Many web-based three-dimensional (3D) structure tools have been developed for displaying existing models, building new models, and preparing initial input files for external calculations. But few of these tools can deal with crystal structures.

**Results:**

We developed a user-friendly and versatile program based on standard web techniques, such as Hyper Text Markup Language 5 (HTML5), Cascade Style Sheet (CSS) and JavaScript. Both non-periodic organic molecule and crystal structure can be visualized, built and edited interactively. The atom, bond, angle and dihedral in a molecule can be viewed and modified using sample mouse operations. A wide range of cheminformatics algorithms for crystal structure are provided, including cleaving surfaces, establishing vacuum layers, and building supercells. Four displayed styles, namely “Primitive cell”, “Original”, “In-cell” and “Packing” can be used to visualize a unit cell. Additionally, the initial input files for Vienna Ab-initio Simulation Package (VASP) and Gaussian can be obtained by interacting with dialog boxes in 3DStructGen.

**Conclusions:**

3DStructGen is a highly platform-independent program. It can provide web service independently or can be integrated into other web platforms. Other than local desktop software, it does not require any additional effort to install the system but a web browser supporting HTML5. 3DStructGen may play a valuable role in online chemistry education and pre-processing of quantum calculations. The program has been released under MIT open-source license and is available on: https://matgen.nscc-gz.cn/Tools.html. 
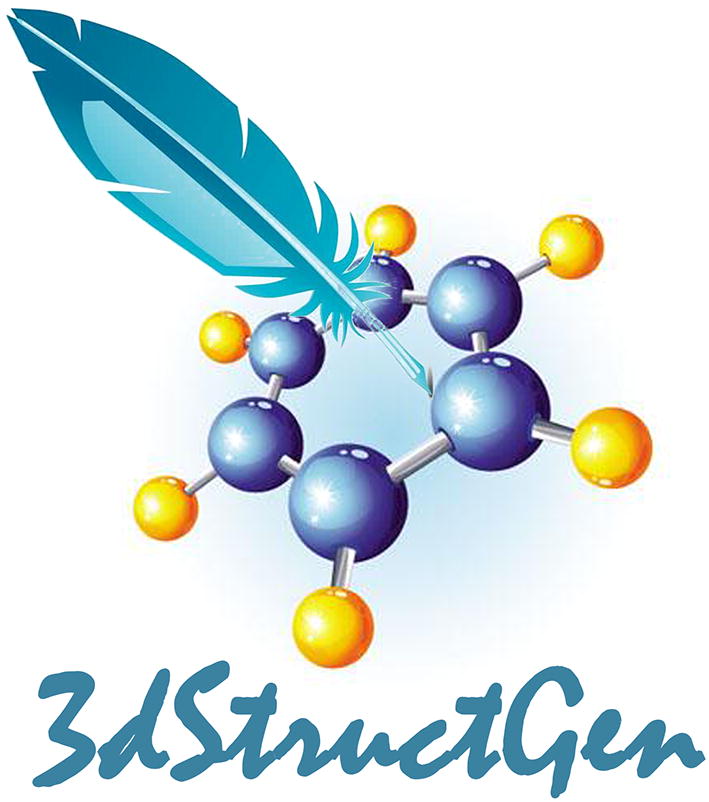

## Background

Molecular visualization plays a vital role in constructing databases and repositories, preparing initial structures for quantum and molecular dynamics calculations, and analyzing the trajectory coordinates in real time. Visualizing and interactively editing the structure of the molecules can effectively reduce the simulation threshold and improve efficiency. Recent development in the field of high-performance computing (HPC) and cloud computing has led to many online computing platforms. For instance, MatCloud [1], rescale [2], WebMO [3], and MolCalc [4], provide web services for materials and chemistry calculation. These web-based tools provide remotely controlling computing resources, and their accessibility and compatibility make them extremely popular. Nowadays, it is common to use the web-based 3D structural visualization and editor tools to generate the model structures and prepare the input files for simulations.

The state-of-the-art visualization software in the field of computational chemistry is mainly developed on desktop environment, such as Avogadro [5], Materials Studio [6], and VESTA [7]. With the increasing demand for web-based visualization, several relevant tools have emerged. For instance, 3Dmol [8], JSmol [9] and Web3DMol [10] contain powerful visualization capabilities, but the structure editing function is missing. Another tools, such as ChemdoodleWeb [11], Kekule.js [12], JSME [13], can only edit 2 dimension (2D) structures. 3D structure editing tools (e.g., ChemMozart [14], CH5M3D [15]) are also reported in the literature. However, these tools focus on organic molecular modeling and do not support processing of crystal structure. In addition, ChemMozart is implemented using node.js framework, and it cannot be used as a lightweight library alone.

In order to process chemical information on web server, we developed an advanced chemical visualization and editor program with the following features. Firstly, both non-periodic organic molecule and crystal structure can be visualized, built and edited interactively. Secondly, a wide range of cheminformatics algorithms for crystal structure are provided, such as cleaving surfaces, establishing vacuum layers, and creating supercells. Thirdly, it is a lightweight library implemented using JavaScript, and it can be easily used to provide web service independently or integrated into other web platforms. Finally, the initial input files for VASP [16, 17] and Gaussian [18] can be generated by interacting with dialog boxes.

### Implementation

**Fig. 1:**
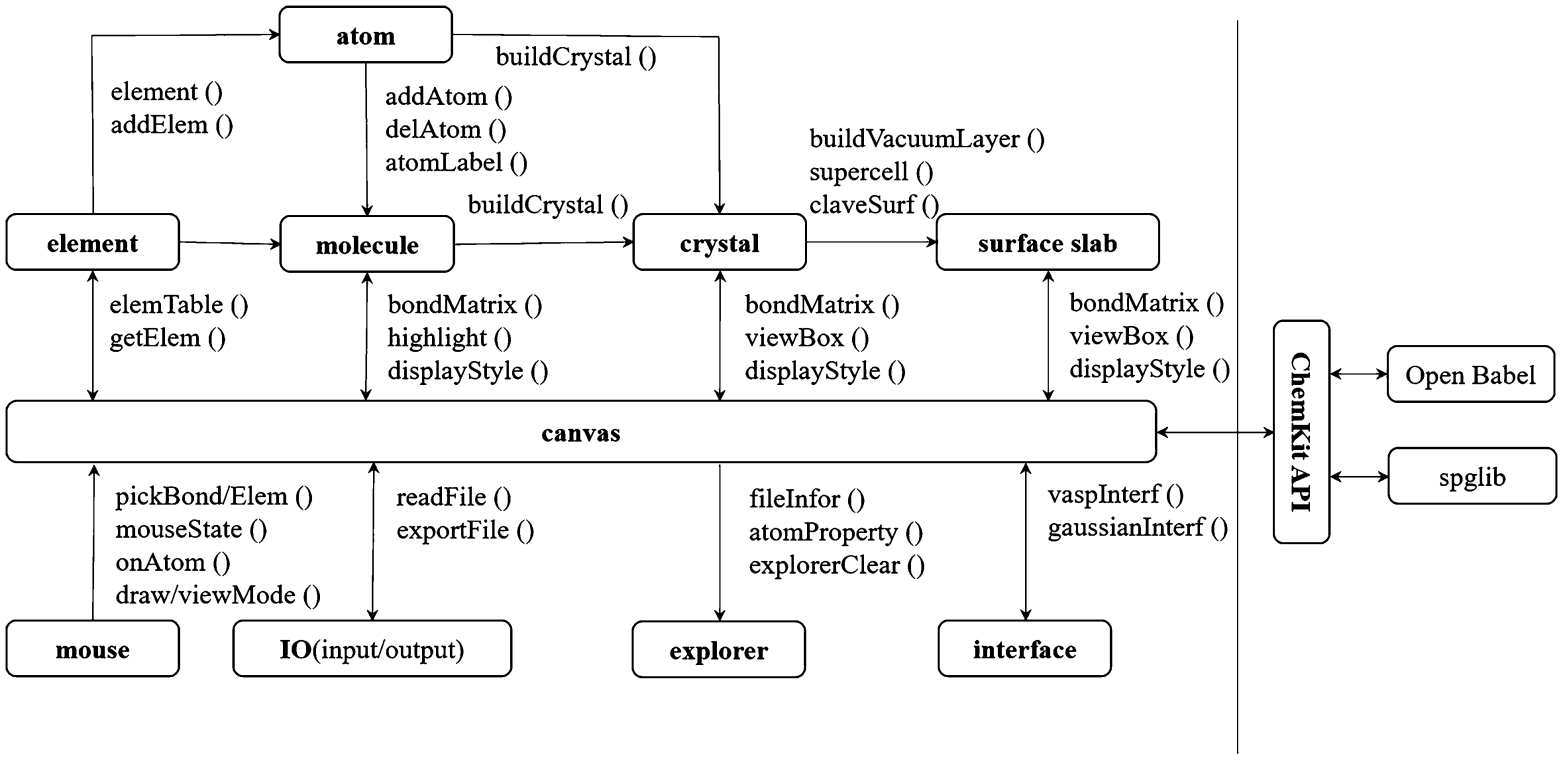
Overview of the core modules and functions in 3DStructGen

Same as most graphical user interface (GUI) programs for computing chemistry [5–7, 19], 3DStructGen focuses on generation of initial molecular geometry. As shown in Fig. 1, the basic modules of molecule, crystal and surface slab consist of several methods to deal with non-periodic molecule, periodic crystal, as well as surface slab system, respectively. The HTML5 canvas is the central module for displaying 3D structures and connecting all other modules. The mouse module provides a number of interactive operations by mouse for users. The processing methods for general chemical file formats (XYZ, SD, MOL, CIF) are supported in IO module, and the initial input file for VASP and Gaussian can be produced using interface module. The explorer module prints the interactive information (e.g., file name, lattice parameter, atom distance, atom coordinate, etc.) for users in real-time. Additionally, ChemKit API provides extensions of 3DStructGen by integrating other cheminformatics tools.

### Element, atom and molecule

The parameters of elements (e.g., atom number, element symbol, atomic mass, color, covalent radii, etc.) are defined as a list in “element ()” function. The “addElem ()” function is used to add an element specified by users, and the corresponding atom model will be visualized on canvas. All elements are displayed in the same color as in the VESTA program. The methane molecule is presented in the canvas when 3DStructGen is initialized. The “ball-and-stick” representation of the structures with the size of atoms based on their covalent radii by default. Besides, the “stick-and-line” models can be specified by users as well.

The “addAtom ()” and “delAtom ()” functions are used to add a new atom and delete an existing atom in a molecule, respectively. By default, a suitable number of hydrogen atoms will be added according to the rule of octet theory, and the bond angles are assigned a value based on orbital hybridization (sp3 in default) selected by users.

**Fig. 2:**
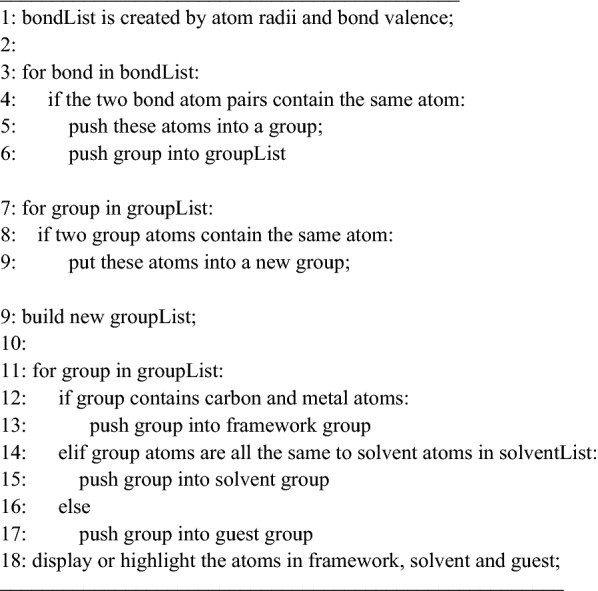
The pseudo code of graph-labeling algorithm

When a new atom is added, a bond is created within its value of the sum of covalent radii of the bonded atoms. The connection between pairs of atoms can be added using mouse operations by calling “addBond ()” function, defined in files or calculated by the 3DStructGen. The “createBond ()” function describes the detailed the bonding rule: firstly, a list of possible bonding atoms based on the bond valance model [20] are built, and then the distance for these possible bonding atoms are calculated. A bond is created if the distance falls between 0.5 and 1.2 times the sum of the covalent radii for these bonding atoms. Here, the 0.5 times distance is used to avoid bonding between the closed atoms (maybe overlap atoms). The “bondMatrix ()” function defines a public routine to store and return the bond list. The graph-labeling algorithm [21] (pseudo code in Fig. 2) used for recognizing framework and guest (including solvent molecule) are developed to deal with metal–organic frameworks (MOF) in Cambridge structural database (CSD) database. Our bonding method has shown satisfactory results for this task, and the visualization results can be found in the “Structure visualization” section.

When a structure is created, an atom or a group of atoms selected by users can be moved in up, down, left and right directions by a user-specified distance. A crude geometry optimization is supported for non-periodic molecule by optimizing the bond lengths, bond angles and dihedral following the same method in CH5M3D [15]. An optional geometry optimization based on force filed method is also provided by the using ChemKit API, which is explained in further details in the “ChemKit API” section.

### Crystal and surface slab

The “buildCrystal ()” function is used to build crystal lattice structure. By clicking the dialog box of “build crystal”, the symmetry space groups (230 in total), lattice parameters, atom types and coordinates can be interactively selected or edited by users. When all the operations are finished, a crystal structure will be shown in the canvas.

The supercell slab is a structural model used in quantum or molecular dynamics simulations to study the surface kinetic [22], thermodynamic [23], and electronic [24] properties. The “cleaveSurf ()” function shows an algorithm to construct surface slab with any Miller index orientation from a bulk unit cell of any Bravais lattice. A slab with two surfaces is embedded with vacuum regions in a supercell. Sun [25] proposed an efficient algorithm for constructing slab surface, and the same method is used to find two basis vectors that span a given surface orientation, $\varvec{v}_{1}$ and $\varvec{v}_{2}$. The followings are the detailed descriptions:

If a Miller index contains no zeros, we take (hkl) as an example:

1 $$\begin{aligned} & \varvec{p}_{1} = \left( {M/h} \right){\mathbf{a}} \\ & \varvec{p}_{2} = \left( {M/k} \right){\mathbf{b}} \\ & \varvec{p}_{3} = \left( {M/l} \right){\mathbf{c}} \\ \end{aligned}$$

If a Miller index contains one zero, we take (hk0) as an example:

2 $$\begin{aligned} & \varvec{p}_{1} = \left( {M/h} \right){\mathbf{a}} \\ & \varvec{p}_{2} = \left( {M/k} \right){\mathbf{b}} \\ & \varvec{p}_{3} = \varvec{p}_{1} + {\mathbf{c}} \\ \end{aligned}$$

If a Miller index contains two zeros, we take (h00) as an example:

3 $$\begin{aligned} & \varvec{p}_{1} = \left( {0, 0, 0} \right) \\ & \varvec{p}_{2} = {\mathbf{b}} \\ & \varvec{p}_{3} = {\mathbf{c}} \\ \end{aligned}$$

Where the **a**, **b** and **c** are [100], [010] and [001], respectively. The $M$ is the least common multiple of h, k and l. Then we get the $\varvec{v}_{1}$ and $\varvec{v}_{2} \varvec{ }$ by:

4 $$\begin{aligned} & \varvec{v}_{1} = \varvec{p}_{1} - \varvec{p}_{1} \\ & \varvec{v}_{2} = \varvec{p}_{3} - \varvec{p}_{1} \\ \end{aligned}$$

If we define the $\varvec{v}_{1} = \left( {v_{11} , v_{12} , v_{13} } \right),$ $\varvec{v}_{2} = \left( {v_{21} , v_{22} , v_{23} } \right)$ and $\varvec{v}_{3} = \left( {v_{31} , v_{32} , v_{33} } \right)$, then we get the third Bravais lattice vectors $\varvec{v}_{3}$ that is maximally orthogonal to these two basis vectors by:

5 $$\begin{aligned} & v_{31} = v_{12} *v_{23} - v_{13} *v_{22} \\ & v_{32} = v_{13} *v_{21} - v_{11} *v_{23} \\ & v_{31} = v_{11} *v_{22} - v_{12} *v_{21} \\ \end{aligned}$$

When the three Bravais lattice vectors of $\varvec{v}_{1}$, $\varvec{v}_{2}$ and $\varvec{v}_{3}$ are determined, the surface-oriented basis transformation is performed and we can get surface slab by these vectors.

**Fig. 3:**
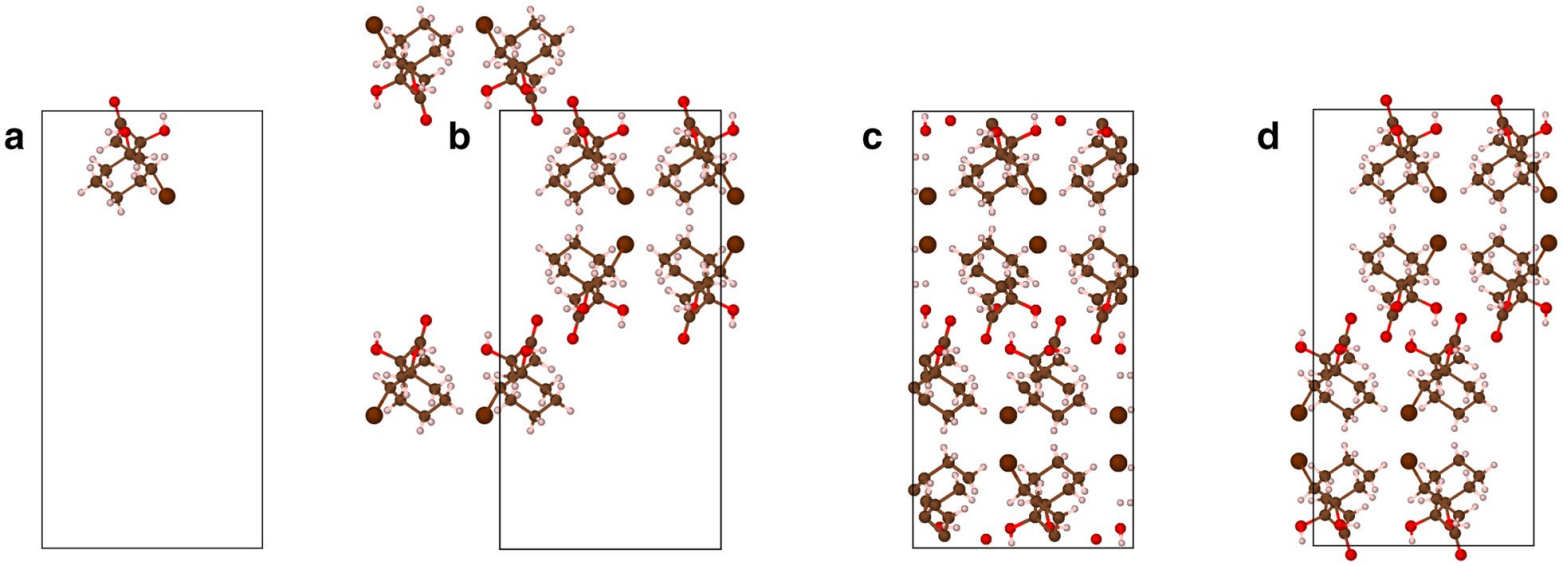
A crystal structure (CSD code: WAJZUE) is depicted in different styles. **a** asymmetric style; **b** original style; **c** in-cell style; **d** packing style. This structure is given in Additional file 1

For a crystal structure composed of a unit cell, and a set of atoms are arranged in a way that the atoms are periodically repeated in three dimensions on a lattice. The “displayStyle ()” function provides several methods to display the repeated atoms, and the relevant images are shown in Fig. 3. The following options can be chosen in the 3DStructGen:

- “Asymmetric”: The minimal subunit of a crystal is presented, consisting of one or more atoms, ions, or molecules, of which geometric arrangement is not related by crystallographic symmetry.
- “Original”: Using this style, each atom will be displayed in its original location, together with copies formed by applying each of the operators of the symmetry group.
- “In-cell”: The atoms are displayed based on whether their coordinates, as well as their symmetric copies are in the lattice cell.
- “Packing”: The complete molecules and their symmetric copies in a lattice cell will be displayed depending on the geometric center of connected sets of atoms. This gives a display of conventional cell for most types of crystal system.

The above styles allow users to visualize atoms in desired manners for different scenarios. The “Asymmetric” may be useful to focus on details of the minimal structure when all the copies are invisible, especially for large and complex models. The “Original” and “Packing” styles provide the copies of units, which demonstrate symmetric information of a crystal. The “In-cell” style catches a glimpse of all possible atoms in the lattice cell, which matches the real scene of truncating a lattice cell from the whole periodic system.

## 2D canvas and mouse

The HTML5 canvas is a revolutionary technology for graphics and visualization on the web. Powered by JS, the HTML5 Canvas API allows 3DStrcutGen to create visualizations and animations of 3D structures on a 2D canvas in the browser without additional plugins. The <canvas> tag creates a bitmapped surface to be drawn and viewed in a HTML document. Although the canvas element is 2D, atoms are sorted by depth and drawn from back-to-front to show 3D effect.

Users can interact with the canvas using mouse. The functions of “mouseMove ()”, “mouseWheel ()”, “mouseDown ()” and “mouseUp ()” are designed to respond to the corresponding mouse events triggered by users. The separated “display mode” and “edit mode” have different mouse events. In display mode, the structure in the canvas can be rotated, translated and the information of atom, bond and angle can be selected to display. While in the edit mode, the structure can be edited. For instance, the “onAtom ()” function returns whether the mouse is within some distance to the selected atom. If the function returns “true”, this atom will be assigned.

### IO and explorer

3DStructGen implements a wide range of cheminformatics algorithms for handling chemical data, including non-periodic molecule and crystal structure. For non-periodic molecule, several file formats (XYZ, SD and MOL) are supported in 3DStructGen. Note that the CIF files obtained from different database or exported by different software may contain minor difference, which can lead to unsuccessful data reading. We have performed a comprehensive test using data from inorganic crystal structure database (ICSD) [26], Cambridge structural database (CSD) [27], crystallography open database (COD) [28] and Materials Studio software.

The explorer module contains a system window to display the imported file information (e.g., file name, lattice size, formula, etc.), and a property window to print the structure information (e.g., atom coordinate, bond distance, angle size, etc.) when a user interacts with the canvas.

### Calculation interface

In order to facilitate the quantum calculation, we developed the input file generation interfaces for quantum calculation software, including VASP and Gaussian. For VASP, the initial files of INCAR and KPOINTS can be created online by setting the corresponding parameters in the dialogs, and the information of atoms and lattice of the existed crystal structure in the canvas is written into POSCAR. Gaussian is a popular alternative for non-periodic molecules, and only one input file needs to be prepared. Similarly, a dialog is designed for setting parameters and a file named “Gaussian.gif” will be generated when all interactive operations are performed. It should be noted that the calculation interface is independent to 3DStructGen except for structure information. Thus, the other calculation interfaces can be easily added into this program.

### ChemKit API

ChemKit API is a RESTful web service that wraps the cheminformatics tools of Open Babel [29] and spglib [30], and expands the capacity of the 3DStructGen. Open Babel is a popular chemical toolbox to handle non-periodic molecule structures, and the spglib is a useful tool for finding and processing crystal symmetries. The ChemKit API is not a part of the 3DStructGen, but an optional tool. The following code shows an example of optimizing the molecule geometry in the canvas using Open Babel:
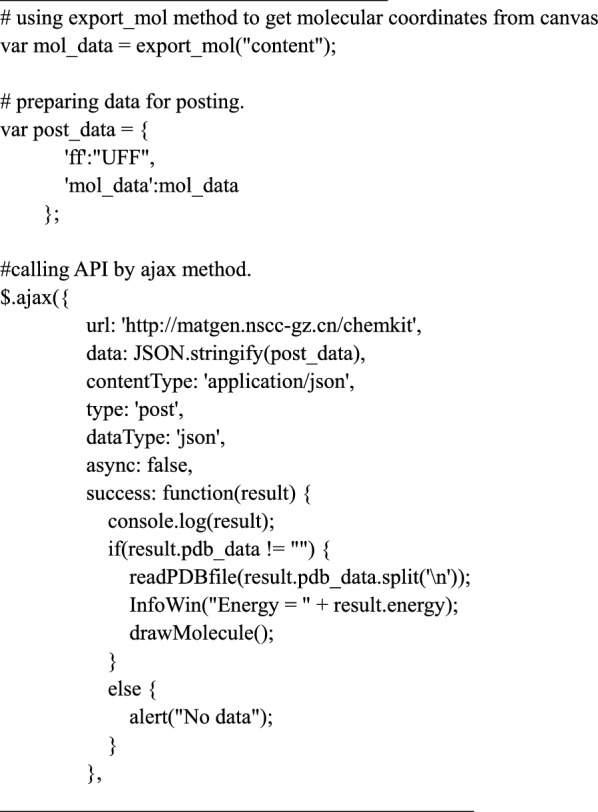


### Installation

The core of this program is written in JavaScript, which has no special requirement for installation but a web browser supporting HTML5. This package has been extensively tested with recent versions of the most commonly used browsers, namely, Microsoft Internet Explorer (v10), Mozilla Firefox (v19), Google Chrome (v70) and Opera (v12). The ChemKit API is an optional tool for 3DStructGen, which can be used when the local devices connect to the internet.

## Result and discussion

### User interface

**Fig. 4:**
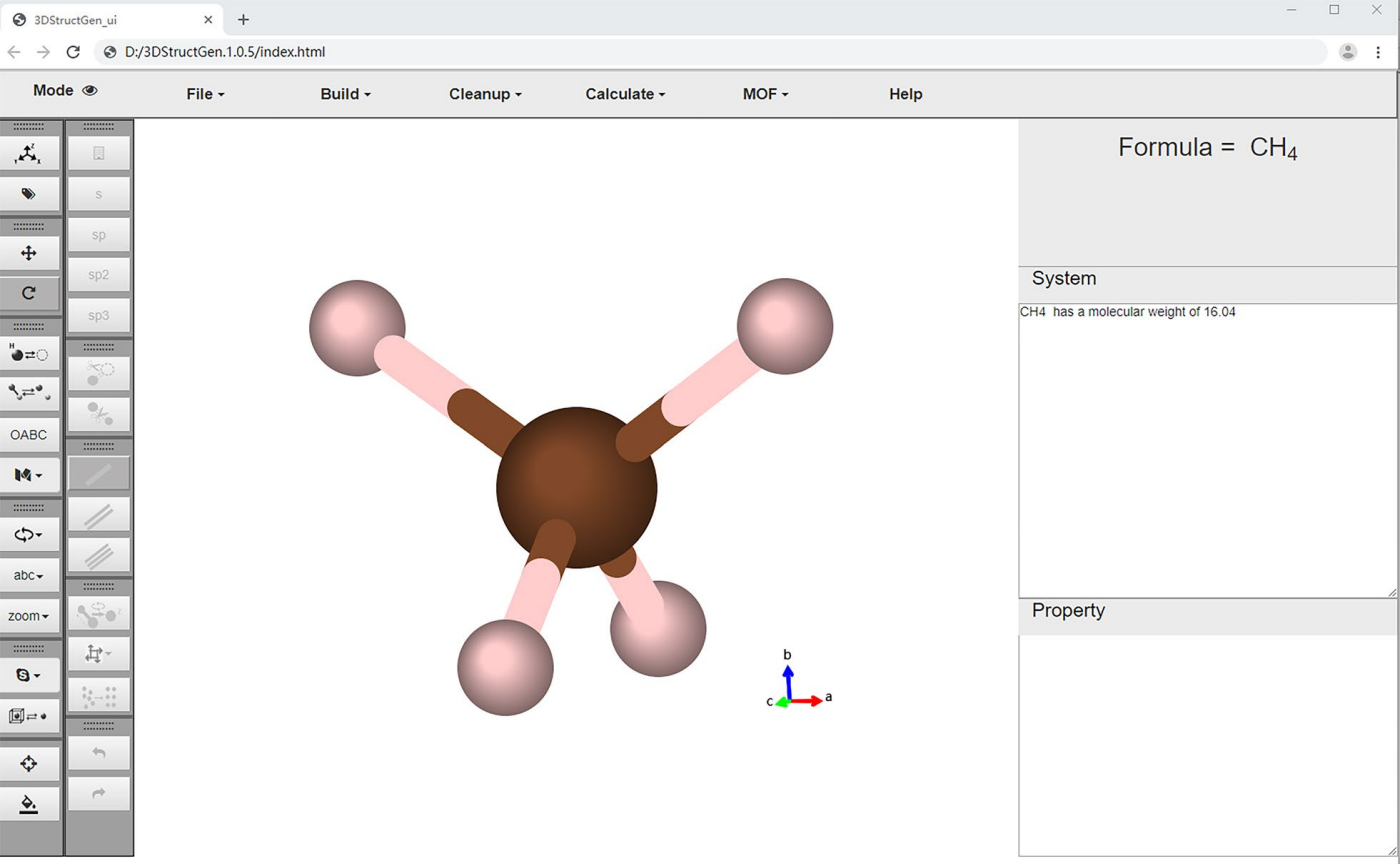
The initial user interface of 3DStructGen

Figure 4 displays the user interface of the 3DStructGen. On the left side of the canvas, there are the dialog buttons for viewing and editing the structure interactively. The separate “display” and “edit” modes can be switched by users, and the corresponding dialog buttons will be activated or deactivated accordingly. The top side of the canvas is the functional zone, where users can choose to load a new file, build complex crystal structure, clear up canvas or system window, prepare the initial input files for quantum calculation, and perform other functions. The system and property windows are showed on the right side of canvas. The new file information (e.g., file name, structure formula, lattice parameters, etc.) will be printed on the system window, while the structure information, including atom types, coordinates, bond distance, angle size will be displayed on the property window.

### Structure visualization

**Fig. 5:**
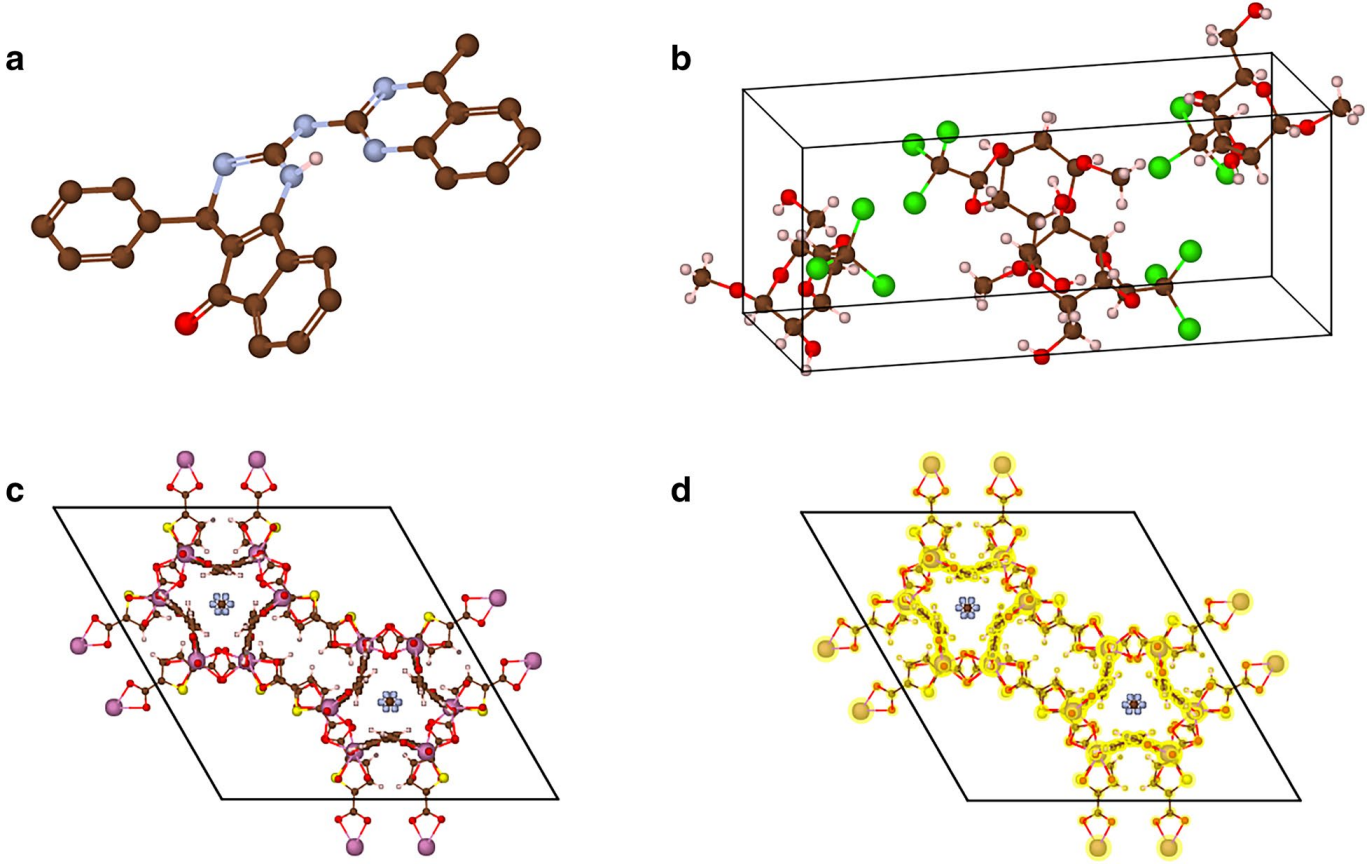
The images of a non-periodic molecule and organic crystal structure. **a** An organic molecule containing double bonds; **b** organic crystal structure (CSD code: PONCOL) displayed with “Packing” style. **c** metal–organic framework structure (CSD code: ABEXEN) displayed with “Packing” style. **d** The framework molecule is highlighted with yellow color. The organic crystal structures can be found in supplement (see Additional file 1)

In order to demonstrate the capacities of our program, a number of snapshots generated by 3DStructGen has been provided. Organic molecules are the most commonly used structures in the field of cheminformatics. Figure 5 shows an illustration of a non-periodic molecule with double bonds (Fig. 5a) and organic crystal structure (Fig. 5b) in the canvas of 3DStructGen, respectively. The Metal–Organic Framework is a specific structure in chemistry, Fig. 5c illustrates an example of Metal–Organic Framework structure, and Fig. 5d shows the highlighted framework molecules with yellow color.

**Fig. 6:**
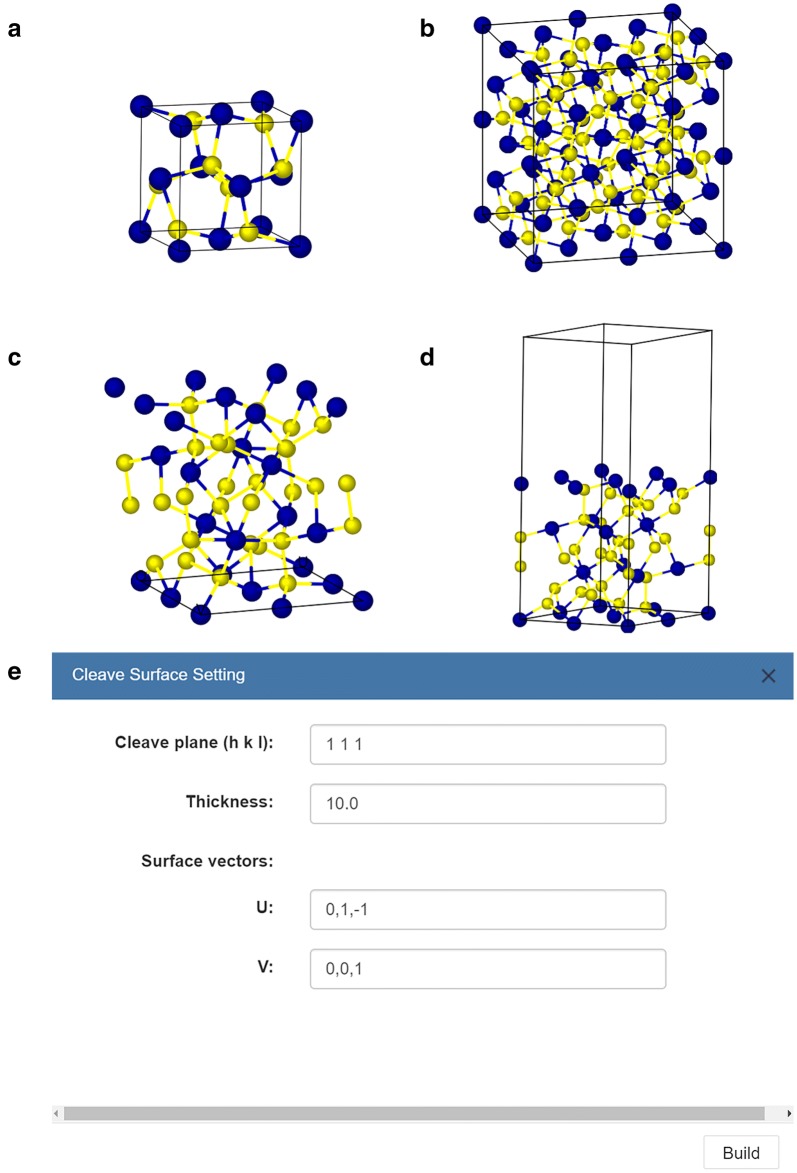
The inorganic crystal structures representations. **a** Inorganic crystal structure of CoS_2_ (ICSD code: 86351); **b** supercell model with 2 × 2 × 2. **c** Cleaving the unit cell with (111) surface. **d** Constructing the (111) surface slab of CoS_2_ with a 10 vacuum. **e** The dialog box of “cleave surface”. The CoS_2_ structure is given in Additional file 1

Figure 6 shows another example of inorganic compound of CoS_2_. The unit cell of CoS_2_ and its supercell of 2 × 2 × 2 are depicted in Fig. 6a, b, respectively. Figure 6c shows the (111) surface slab with a 10 Å thickness. The two basis vectors of (− 1, 1, 0) and (− 1, 0, 1) that span a given surface orientation are automatically generated by 3DStructGen when the vector of cleaving plane has been conformed (seeing dialog box in Fig. 6e). Finally, a new crystal structure with 10 Å vacuum layer has been created in Fig. 6d.

### Interface for preparing quantum calculation input files

When the simulation structure has been built on the canvas successfully, the dialog of Quantum interface can be used to prepare the input files for the calculation software. To allow convenient submission of computing tasks and reduce the time cost of the calculation preparation stage, *3DStructGen* provides two kinds of quantum interface for Gaussian and VASP (i.e. *.gjf file for Gaussian and *INCAR, POSCAR* and *KPOINTS* file for VASP), respectively. In this section, the Gaussian interface will be introduced in detail.

**Fig. 7:**
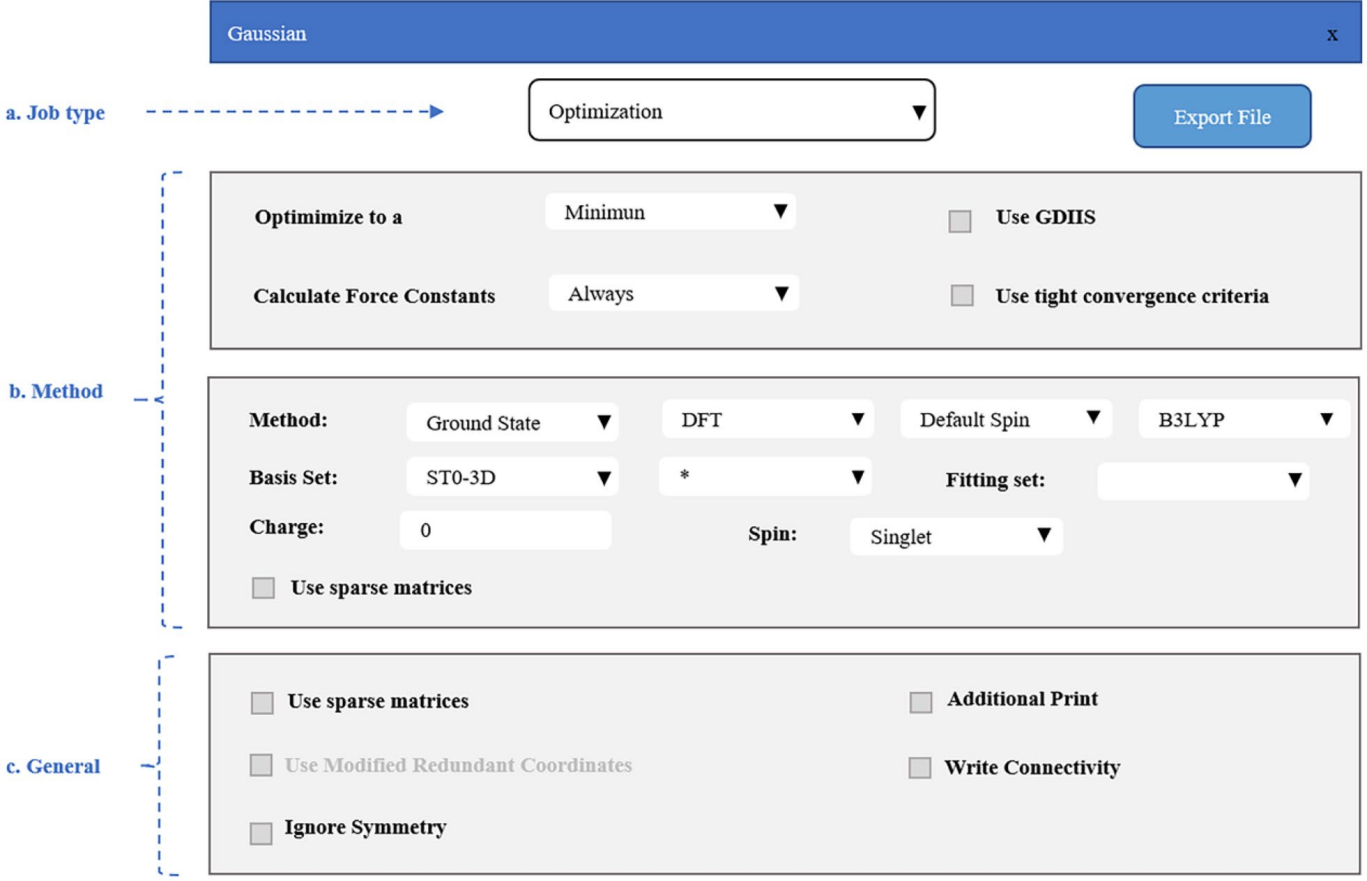
The snapshot of dialog box of Gaussian interface and relevant functional parts in *3DStructGen*


The Gaussian interface is mainly composed of three parts: Job type, Method, and General, which are corresponding to the part *a*, *b* and *c* in Fig. 7, respectively. Firstly, different types of tasks are available for selection in the Job type section. In addition to the *Optimization* module shown in the figure, the section also contains *Energy*, *Frequency*, *Opt *+ *Freq*, *IRC*, *Scan*, *Stability*, *NMR*, *BOMD* and *ADMP* modules, which cover almost all basic computing task types. After a specific task type is selected, Method section shows an interface of the more explicit parameter settings for calculation. The different job types offer different parameter settings option. For instance, in *Optimization* module, the calculation method, basic set used, spin and charge can be chosen based on user specific molecule model and simulation demand. Here, we chose the DFT method and 3-21G basis set, and set the exchange–correlation to B3LYP and the other options to the respective default values. Finally, several general settings can be tuned in the General section, such as *Use Quadratically Convergent SCF*, *Ignore Symmetry*, *Write Connectivity* etc., when the required calculation parameters are confirmed, one can click the *Export file* button to obtain the Gaussian calculation input file generated by 3DStructGen automatically. The following shows the content of an exported file:

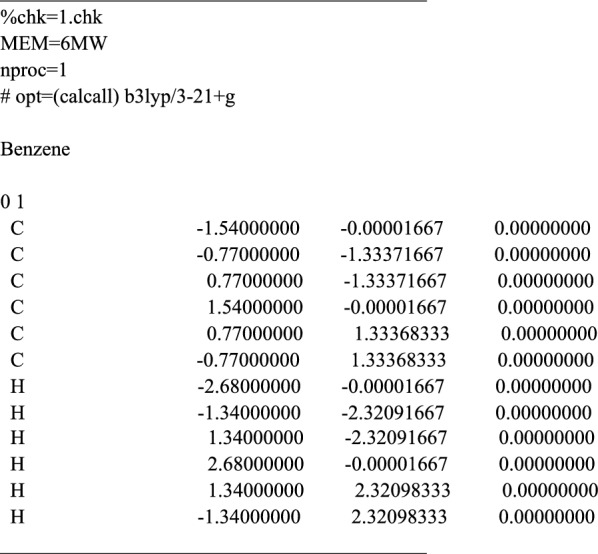


This process of *3DStructGen* will greatly facilitate users to prepare their calculation tasks.

### Comparisons with other web-based visualization programs

For an advanced web-based visualization program, displaying and editing the structure in 3D are crucial. As shown in Table 1, all programs support 3D visualization but JSME, which is a 2D molecule editor facilitating graphical input and interactive editing of molecules. JSmol and 3Dmol.js are the pure 3D representation of molecular structure. The 2D technology can deal with non-periodic molecule. Examples are Chemdoodle and Kekule.js, which contain a separate 2D editor for creating the initial structure and viewing the structure on 3D canvas. But these methods are inoperative for periodic crystal. As a result, combining the displaying and editing function into one canvas maybe a promising method, and the WebMO, Marvin.js, Chemozart, CH5M3D belong to this category. WebGL is an advanced technology by using hardware-acceleration, which allows larger molecules to be visualized, provides smoother interaction, and produces higher-resolution picture. The WebMO and Chemozart are good examples equipped with WebGL. However, Chemozart is still in its initial version and only support for non-periodic molecules. WebMO satisfies all the previous conditions, but it requires commercial license and it is not open source for academic users. 3DStructGen supports for the 3D visualization and editing of non-periodic molecule, crystal and surface slab, and the source code is free to users, which is still competitive compared with other web-based visualization programs. The initial version of 3DStructGen focuses on developing general functions, preparing the input files for the quantum calculations. The implementation of WebGL is desirable and will be considered in a future version.

**Table 1:** Comparisons of the popular web-based visualization programs

Program	Descriptions				License	Published time
	3D visualization	3D editor	WebGL	Support structure types		
3DStructGen	√	√	×	ABC	MIT	This paper
Chemdoodle	√	×(2D)	√	AB	GPL	2015
JSmol	√	×	√	AB	GPL	2001
WebMO	√	√	√	AB	Commercial	2000
Marvin.js	√	√	×	A	Commercial	–
Chemozart	√	√	√	A	Apache2.0	2015
Web3DMol	√	×	√	A	MIT	2017
3Dmol.js	√	×	√	A	BSD	2015
Kekule.js	√	×(2D)	×	A	MIT	2016
CH5M3D	√	√	×	A	GPL	2013
JSME	×	×(2D)	×	A	BSD	2013

Structure type (A: non-periodic molecule; B: crystal; C: surface slab)

### Extensions for web-based tools

Comparing to local desktop packages, the complex software system for web-based tools in the field of cheminformatics is still limited by webpage programing language and data transmission. For instance, the Open Babel library was originally written in C++, and the author of Kekule.js tried to compile the Open Babel into JavaScript by Emscripten to support more chemical file formats and molecule force field calculation [12]. However, the JavaScript version Open Babel is about 10 MB, and a long time is needed to transfer all the data over the Internet. Thus, a RESTful web service wrapping the cheminformatics tools maybe a practical solution. The ChemDoodle tool gives an example of accessing the ChemDoodle desktop API by using iChemLabs cloud services for additional tools [11]. In our program, we also provide a free ChemKit API for expanding the capacity of web-based tools.

## Conclusions

We introduced a user-friendly and versatile program to view, build and edit non-periodic organic molecule and crystal structure using standard web techniques, such as HTML5, CSS and JavaScript. The improved capacity for crystal structures expands scope of applications in the field of cheminformatics. It is well designed with numerous functional modules and flexible enough to integrated with another program. The highly platform-independent nature allows 3DStructGen to be easily installed, requiring no additional software or any special permissions. This program is released under MIT license, which is free and open source for users. We hope that the 3DStructGen can play a vital role in online chemistry education and pre-processing of quantum calculations.

## Supplementary information

**Additional file 1.** The structure files used in this article.

## Data Availability

The source code of this program is available for download from page https://matgen.nscc-gz.cn/Tools.html. Additional file 1 covers the structure files used in this article.
